# Targeting senescence to prevent diabetic kidney disease: Exploring molecular mechanisms and potential therapeutic targets for disease management

**DOI:** 10.1111/dme.15408

**Published:** 2024-07-12

**Authors:** Paige Charlotte Alison Phillips, Mafalda de Sousa Loreto Aresta Branco, Chelsy Louise Cliff, Joanna Kate Ward, Paul Edward Squires, Claire Elizabeth Hills

**Affiliations:** ^1^ Joseph Banks Laboratories, College of Health and Science Lincoln UK

**Keywords:** diabetes, diabetic kidney disease, diabetic nephropathy, senescence, senescence‐associated secretory phenotype, SGLT2i, inflammation

## Abstract

**Background/Aims:**

As a microvascular complication, diabetic kidney disease is the leading cause of chronic kidney disease and end‐stage renal disease worldwide. While the underlying pathophysiology driving transition of diabetic kidney disease to renal failure is yet to be fully understood, recent studies suggest that cellular senescence is central in disease development and progression. Consequently, understanding the molecular mechanisms which initiate and drive senescence in response to the diabetic milieu is crucial in developing targeted therapies that halt progression of renal disease.

**Methods:**

To understand the mechanistic pathways underpinning cellular senescence in the context of diabetic kidney disease, we reviewed the literature using PubMed for English language articles that contained key words related to senescence, inflammation, fibrosis, senescence‐associated secretory phenotype (SASP), autophagy, and diabetes.

**Results:**

Aberrant accumulation of metabolically active senescent cells is a notable event in the progression of diabetic kidney disease. Through autocrine‐ and paracrine‐mediated mechanisms, resident senescent cells potentiate inflammation and fibrosis through increased expression and secretion of pro‐inflammatory cytokines, chemoattractants, recruitment of immune cells, myofibroblast activation, and extracellular matrix remodelling. Compounds that eliminate senescent cells and/or target the SASP – including senolytic and senomorphics drugs – demonstrate promising results in reducing the senescent cell burden and associated pro‐inflammatory effect.

**Conclusions:**

Here we evidence the link between senescence and diabetic kidney disease and highlight underlying molecular mechanisms and potential therapeutic targets that could be exploited to delay disease progression and improve outcomes for individuals with the disease. Trials are now required to translate their therapeutic potential to a clinical setting.

**What's new?**

- This brief review summarises the implications of renal senescence in the context of diabetic kidney disease (DKD), utilising recent articles that explore molecular mechanisms that underpin its induction and the role of the pro‐inflammatory secretome in exacerbating disease progression.
- We specifically review the role of senescent cell clearance through various exogenous and endogenous protectors and discuss the clinical relevance of reducing the senescent cell burden as a strategy to slow disease progression in DKD.

## 1 INTRODUCTION

Initially characterised in human diploid fibroblasts, senescent cells were described as having a limited replicative potential with irreversible cell cycle arrest after serial cultivation.^1^ Cessation of cell turnover generally occurs in the G1 phase,^2^, ^3^ with three broad forms of senescence recognised: (i) telomere attrition‐induced senescence,^4^ caused by telomere shortening as a result of cellular replication, (ii) oncogene‐induced senescence, which refers to suppression of cellular proliferation in response to activation of oncogenic signalling^5^ and (iii) stress‐induced senescence, which is attributed to injury stimuli such as oxidative stress,^6^ DNA damage^7^ and high glucose,^8^, ^9^ and occurs independently of telomere length.

**FIGURE 1:**
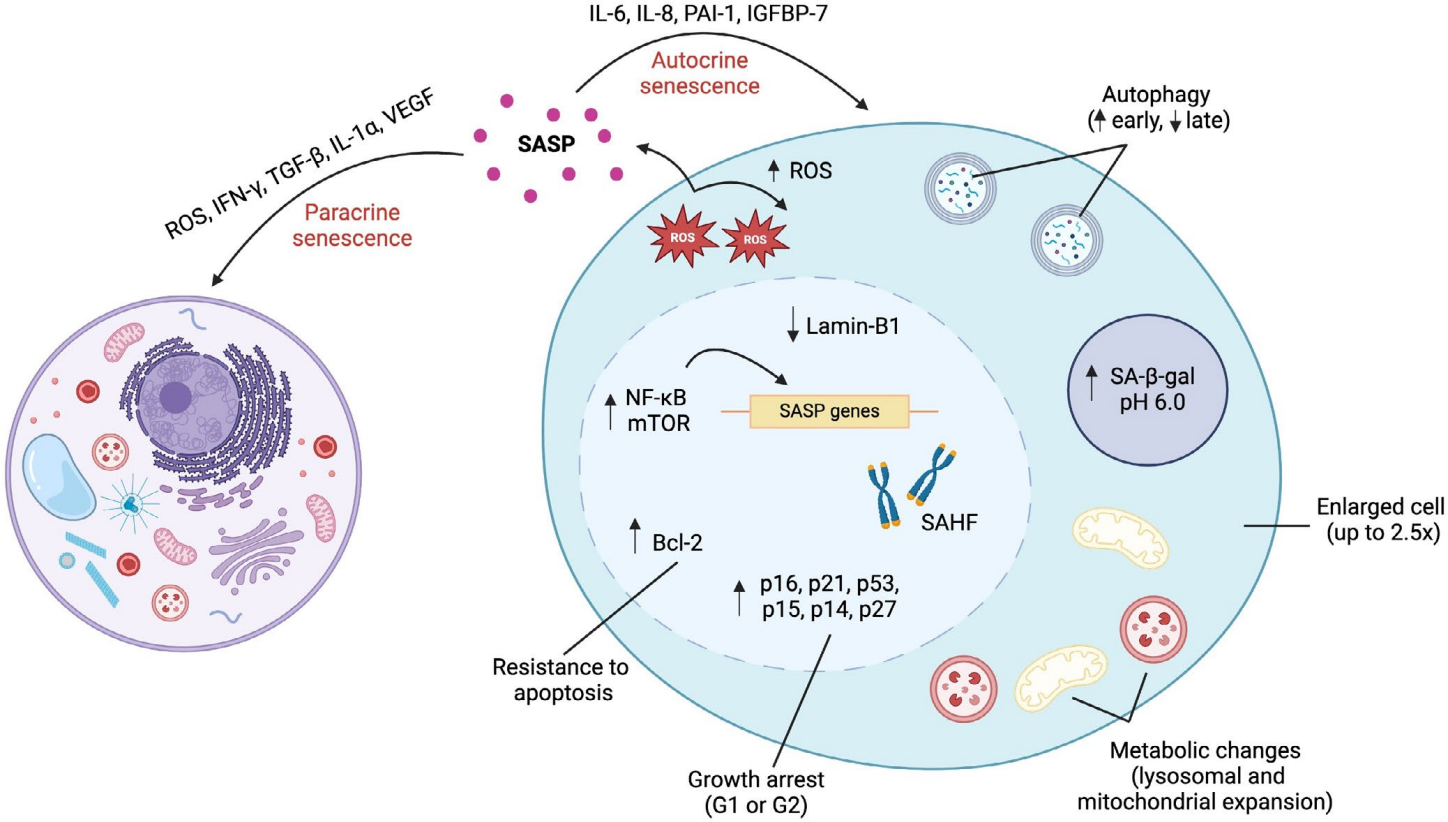
General molecular hallmarks of cellular senescence. Evaluation of multiple markers of senescence is required in the classification of cell senescence. Those commonly used include up‐regulation of cell cycle inhibitors (e.g., p16, p21 and p53), elevations in β‐galactosidase activity (measurable at pH 6.0), and common morphological alterations (e.g., irregular nuclear formation as a consequence of the loss of lamin‐B1 and cellular enlargement due to restricted proliferation but continued growth). Due to dysfunctional mitochondria, senescent cells generate elevated levels of reactive oxygen species. These reinforce irreversible cell cycle arrest through activation of the DNA damage response pathway, observations compounded in the presence of increased expression anti‐apoptotic proteins (such as Bcl‐2). Upstream of cell senescence, elevations in mTOR impair the autophagy axis, promoting cell survival in the absence of cellular proliferation. Accumulation of these metabolically active cells impacts on cell function and further senescence through the detrimental effects of the pro‐inflammatory secretome, comprised of both pro‐inflammatory and pro‐fibrotic molecules such as IL‐6, IL‐8, IL‐1α and TGF‐β1. Transcription of these SASP‐associated genes is regulated by NF‐kB, which is referred to as the master regulator of the SASP and is a notable hallmark of senescence due to its notable increase. IFN‐y, interferon gamma; IGFBP‐7, insulin‐like growth factor binding protein 7; IL‐1α, interleukin‐1 alpha; IL‐6, interleukin 6; IL‐8, interleukin 8; mTOR, mammalian target of rapamycin; NF‐kB, nuclear factor kappa B; PAI‐1, plasminogen activator inhibitor‐1; ROS, reactive oxygen species; SA‐β‐gal, senescence‐associated beta galactosidase; SAHF, senescence‐associated heterochromatic foci; SASP, senescence‐associated secretory phenotype; TGF‐β1, transforming growth factor beta; VEGF, vascular endothelial growth factor.

Senescent cells often present with an enlarged, flattened morphology and are accompanied by organellar abnormalities such as irregular nuclei and cytoplasmic granularities.^10^ These cells exhibit increased expression of cell cycle inhibitors (e.g., p16, p21 and p53); have elevated senescence‐associated β‐galactosidase activity (SA‐β‐gal) and are associated with chromatin alteration and reorganisation (e.g., heterochromatin foci).^11^ Moreover, senescent cells display increased expression of anti‐apoptotic/pro‐survival proteins such as B‐cell lymphoma 2 (Bcl‐2) and B‐cell lymphoma‐extra‐large (Bcl‐xL), thereby resisting apoptosis and accumulating at sites of injury^10^ (see: Figure 1).

Usually cleared by the immune system, senescent cells accumulate with age or at the site of tissue damage in response to sustained injury. Although devoid of proliferative capacity, these cells remain metabolically viable and undergo significant metabolic reprogramming, ensuring they retain their growth‐arrested state and express the genes and proteins required to sustain the highly complex, dynamic and variable ‘senescence‐associated secretory phenotype’ (SASP).^12^ Traditionally considered to exhibit increased glycolysis, repressed autophagy and abnormal lipid metabolism, the picture is complex; with cellular senescence tightly orchestrated by a number of different metabolic inducers and alterations involved in cellular metabolism.^13^ Initiation of senescence is heterogeneous and occurs within multiple contexts throughout the normal lifespan and across different tissue types. Acute senescence is a physiologically appropriate and tightly orchestrated biological process that occurs in response to cell extrinsic stimuli (e.g., injury, cancer and DNA damage) to maintain organogenesis and tissue homeostasis.^14^ In this context, senescent cells play an important role in both wound healing and tissue repair and are cleared in a timely manner by macrophages and natural killer cells as part of the innate immune response.^14^ Conversely, chronic senescence refers to the dysregulated accumulation of senescent cells which, although usually cleared by the immune system,^14^ accumulate with age and in response to disease, resisting apoptosis and presenting with increased SA‐β‐gal activity and enhanced expression of cell cycle inhibitors.^15^ These cells are implicated in the development of inflammation and fibrosis by limiting tissue rejuvenation and secretion of pro‐inflammatory and pro‐fibrotic mediators designated as the SASP.^12^ Despite its fundamental role in defence against infection or insult, an exaggerated and/or prolonged inflammatory response can be detrimental to health. Consequently, disease prevalence in the elderly is greater than in the general population,^16^ with age being one of the strongest risk factors associated with multiple chronic inflammatory conditions.^17^, ^18^, ^19^ These events help explain why, despite an increasing lifespan in the general population, the corresponding increase in health‐span lags behind.^20^

Compounded by the ageing process, the prevalence of type 2 diabetes mellitus (T2DM) and associated co‐morbidities continue to rise.^21^ Individuals with T2DM display an accelerated ageing phenotype which is characterised by chronic and sterile inflammation.^22^ Accumulation of senescent cells and its SASP have been implicated in the pathology of a wide variety of age‐related diseases,^23^, ^24^, ^25^, ^26^ including diabetes and its secondary complications, for example, impaired wound healing,^27^, ^28^, ^29^ retinopathy,^30^, ^31^, ^32^ neuropathy,^33^ cardiomyopathy^34^, ^35^ and nephropathy.^36^ While hyperglycaemia accelerates cellular senescence, the number of pathways by which this acceleration occurs is extensive and appears to vary between different cell types. Consequently, with no unifying model accounting for hyperglycaemia‐associated senescence, we need to better understand how common mechanisms that underpin multisystem damage triggered by hyperglycaemia, hyperlipidaemia and high blood pressure (i.e., hallmarks of T2DM and cardiometabolic syndrome) are also compounded with advancing age.

## 2 SENESCENCE AND DIABETIC KIDNEY DISEASE

Diabetic kidney disease (DKD) affects around 30–40% of individuals with diabetes^22^ and is associated with an increased risk of cardiovascular disease (CVD)^22^ as well as being the leading cause of end‐stage renal failure worldwide.^37^ In the absence of curative options, a four‐pillared approach to the management of DKD is recommended, including the use of blockers of the renin–angiotensin–aldosterone system (RAAS), sodium‐glucose co‐transporter‐2 inhibitors (SGLT2i), non‐steroidal mineralocorticoid receptor antagonists and glucagon‐like peptide (GLP)‐1 receptor agonists.^38^ Despite this plethora of interventions, non‐modifiable risk factors combined with social and environmental determinants of health mean that some individuals naturally progress faster into end‐stage renal disease.^39^ Therefore, adjunct therapeutic approaches to target residual risk—often inflammatory in nature—are required.

Our kidneys are vulnerable to the natural ageing process,^40^ a susceptibility likely attributed to their high metabolic activity, which exposes them to elevated levels of oxidative stress^41^ and chronic low‐grade inflammation.^42^ In individuals over 50 years of age, the human kidney exhibits decreased cortical volume, increased surface roughness, a reduction in nephron number and an increased appearance of renal cysts.^43^ Together these changes impact on health and the elderly often exhibit impaired kidney function with age‐driven histological changes.^43^ Although a reduction in renal function with age is normal, the decline in many older individuals is disproportionate. Of the many changes taking place, three factors are considered critically important: senescence (cellular and biological ageing), immune dysfunction and inflammation.^44^, ^45^ Despite differences in aetiology, senescence, inflammation and fibrosis are common to both ageing and kidney disease and recent evidence suggests that an accumulation of senescent cells correlates to the natural decline in kidney function observed with both age^46^ and in the presence of disease, for example, chronic kidney disease (CKD)^47^and DKD.^36^, ^48^

DKD develops in response to structural and functional disturbances in different regions of the kidney, that is, the renal corpuscle^49^ and the proximal tubules.^50^ Increased cellular senescence has been observed in both podocytes and renal tubular cells in people with type 2 diabetic nephropathy,^48^ with reports demonstrating elevated cellular senescence‐related pathways in people with DKD.^36^ Increased activity of these pathways and a consequent ‘senescence‐related signature’ is associated with a declining glomerular filtration rate (GFR) and increased expression of fibrotic genes when compared with people exhibiting a lower senescence signature.^36^ Similarly, in rodent in vivo models of type 1 diabetes mellitus (T1DM) and T2DM, transition of proximal tubule epithelial cells (PTECs) to a senescent phenotype was reported, confirmed by increased SA‐β‐gal activity and elevated expression of cell cycle inhibitors p16, p27 and p21.^51^, ^52^ Furthermore, extensive tubular cell senescence occurred following acute kidney injury (AKI) in a murine model of diabetes and remained unresolved for up to 28 days post initial injury.^53^ This damage correlated with increasing markers of inflammation and loss of renal function.^53^ Additional studies have reported the effect of hyperglycaemia on the induction of renal senescence,^9^, ^54^, ^55^, ^56^, ^57^ with gene expression knockdown of cell cycle inhibitor p21 attenuating cell senescence in high glucose‐cultured proximal tubules.^54^ Constituting approximately 90% of cortical mass, the renal proximal tubules are the active site of glucose reabsorption, solute secretion, hormone production and metabolic function.^58^

In DKD, renal tubules are highly susceptible to injury and tubulointerstitial fibrosis (TIF), a predictor of kidney failure that develops in response to various morphological and phenotypic changes, including epithelial‐to‐mesenchymal transition (EMT), inflammatory cell infiltration, fibroblast activation and extracellular matrix (ECM) remodeling.^59^ Cells of a senescent phenotype may thereby contribute to kidney damage through activation and recruitment of resident and infiltrating stromal and immune cells, deleterious effects that may be attributable to their pro‐inflammatory secretome.

## 3 THE ROLE OF THE SENESCENCE‐ASSOCIATED SECRETORY PHENOTYPE IN INFLAMMATION AND KIDNEY DISEASE

The SASP is a pro‐inflammatory, bioactive secretome comprising a variety of factors including cytokines, chemokines, proteases and growth factors.^12^, ^60^ The composition of the SASP is dynamic and heterogeneous, dictated by the stimulus and cell type undergoing senescence.^60^ Notably, the SASP can mediate its effects on adjacent cells in a paracrine manner—referred to as the ‘bystander effect’—with the release of inflammatory stimuli inducing further senescence in neighbouring cells and tissues.^61^ Recent studies in multiple models of disease link the senescent bystander effect to paracrine‐mediated cell‐to‐cell communication and induction of multiple pathophysiological pathways, for example, EMT^62^, ^63^ and fibroblast‐to‐myofibroblast differentiation.^64^, ^65^

Although comprised of an extensive catalogue of secretory factors, individual compounds associated with the SASP can be produced by non‐senescent cells, for example, immune cells. While studies must assess multiple parameters when evaluating the degree of senescence and its widespread effects, there are several cytokines and chemokines (including pro‐inflammatory cytokines interleukin (IL)‐6 and IL‐8) which are recognised as some of the most robust and highly conserved features of the SASP linked to sustained and chronic sterile inflammation.^66^ Chemokine signalling reinforces senescence across multiple cell types and also recruits immune cells that contribute to systemic inflammation.^67^ Serum levels of IL‐6 are significantly increased in people with DKD as compared with individuals without disease,^68^ and elevated urinary levels of IL‐8 are associated with reduced GFR and proteinuria, key markers of declining renal function.^69^, ^70^ Other SASP inflammatory cytokines include IL‐1α and IL‐1β.^71^ Active levels of IL‐1β are a consequence of caspase‐1‐mediated cleavage of pro‐IL1β, events triggered in response to assembly of the NOD‐like receptor protein‐3 (NLRP3) inflammasome, a protein complex and principal mediator of sterile inflammation across multiple age‐related pathologies.^72^, ^73^ People with diabetic nephropathy have elevated levels of both IL‐1β and IL‐1α in their serum,^74^, ^75^ with a recombinant human IL‐1 receptor antagonist (BLG‐553902) demonstrating efficacy in abrogating accumulation and deposition of fibrotic markers in PTECs.^71^ Moreover, significant reductions in plasma IL‐1α were observed in individuals with DKD prescribed a 3‐day combined oral course of senolytics, with notable reductions in senescent cell markers reported in both adipose and skin biopsies.^76^

Components of the SASP can be reliably quantified in human plasma,^77^ with case–control studies reporting that circulating, elevated levels of SASP proteins (IL‐6 alone and in combination with IL‐1β), are independent predictors of diabetes incidence.^78^, ^79^ These cytokines signal and influence their local environment and that of distant tissues through the widespread effects of the SASP. Dysregulated inter‐organ communication is supported by observations suggesting that the kidney tubule cell‐released SASP factor osteopontin acts as a causal mediator of AKI‐induced remote acute lung injury,^80^, ^81^ while chronic plasma osteopontin levels are linked to adverse clinical outcomes in individuals with CVD.^82^, ^83^ Elevated with creatinine in people with stable coronary artery disease,^82^ osteopontin is associated with multivessel lesions and a decline in renal function^83^ and is also a recognised molecule mediating cardiorenal syndrome.^84^ When cleaved, osteopontin stimulates macrophage migration and fibroblast activation, events initiated by the SASP protein metalloproteinase‐9 (MMP9),^81^ increased levels of which are linked to the pathogenesis of CVD^85^ and CKD^86^ in T2DM. Pro‐fibrotic mediators (including transforming growth factor beta‐1 [TGFβ‐1]), are central components of the SASP and efficacious drivers of renal fibrosis. Serum TGFβ‐1 levels increase with age,^87^, ^88^ and correlate with declining renal function in humans.^89^ Sustained overexpression of TGFβ‐1 is a hallmark of ageing and is linked to senescence, increased SASP, inflammation and fibrosis.^90^, ^91^, ^92^, ^93^ The role for TGFβ‐1 in renal disease pathology is well established,^94^ with increased activity linked to elevated synthesis of ECM components (e.g., collagen and fibronectin) and impaired degradation; characteristic hallmarks of glomerulosclerosis, TIF and inflammation.^95^ Studies also suggest that TGFβ‐1 positively regulates p21 expression via a p53‐independent pathway,^96^ suggesting a direct role in inflammation through exacerbating senescence and downstream SASP production.

## 4 SENESCENT CELL TYPES CONTRIBUTING TO DKD

### 4.1 Glomerular cell senescence and podocyte loss

Early stages of DKD are characterised by a combination of haemodynamic and metabolic perturbations, namely glomerular changes that underpin hyperfiltration, proteinuria, basement membrane thickening, podocyte loss and mesangial hypertrophy.^97^ Deleterious changes to glomerular filtration capacity (including the limited proliferation of cells as a consequence of senescence) are likely a contributing factor in podocyte effacement^98^, ^99^ and impaired autophagy,^100^ with podocyte senescence linked to impaired autophagic flux and early albuminuria in an in vitro model of T1DM.^98^ Notably, podocytes are one of the primary cell types exhibiting an accelerated senescent phenotype in DKD, with increased expression of senescence and SASP markers observed in both people with DKD^48^, ^101^, ^102^, ^103^ and in murine models of T1DM^104^, ^105^ and T2DM.^106^ Elevations of senescent markers after high‐glucose treatment are also observed in in vitro models utilising podocytes^105^ and mesangial cells,^107^ with p53^101^ and p21^102^ each exhibiting increased expression. Elevated p21 expression in these cells appears to be a consequence of increased mammalian target of rapamycin (mTOR) kinase activity and loss of adenosine monophosphate‐activated protein kinase (AMPK) activation and connexin‐43 expression.^107^ Mechanistically, Chen et al. determined that podocytes with glycogen synthase kinase (GSK)‐3β knockdown (a redox sensitive protein hyperreactive in type 2 glomerular podocytes) exhibit diminished SA‐β‐gal staining and decreased levels of p16, p21 and p53 when cultured in high glucose as compared with control.^108^ Similarly, expression of SASP factors including TGFβ‐1, plasminogen activator inhibition‐1 (PAI‐1) and insulin‐like growth factor binding protein‐3 (IGFBP3) were also decreased when GSK3β was silenced.^108^ These benefits are supported by conditioned media transfer studies in which conditioned media from high glucose and TGFβ‐1‐treated podocytes elicit a synergistic, pro‐senescent effect on healthy, neighbouring podocytes.^105^ Such observations establish a role for SASP‐mediated paracrine signalling, suggesting senescent podocytes can initiate senescence within the kidney in both a paracrine‐ and autocrine‐mediated manner in response to the diabetic milieu. As glomerular injury is one of the earliest events to occur in progression of DKD,^109^ studies exploring the paracrine nature of senescence and the SASP within the glomerular corpuscle are key to our understanding of mechanisms where early intervention may ameliorate initial injury and prevent disease progression.

### 4.2 Proximal tubular cell senescence

Critical to selective tubular reabsorption, high metabolic activity renders the proximal tubules susceptible to glycaemic injury,^55^ with epithelial cells identified as the primary location for renal senescence.^110^, ^111^, ^112^ Proximal tubules in the kidneys of people with diabetic nephropathy display an accelerated senescent phenotype.^48^, ^113^ Similarly, in vivo models of T1DM demonstrate that exposure to hyperglycaemia triggers senescent cell accumulation within the proximal tubules,^53^, ^54^, ^114^ observations further supported by studies utilising in vitro models of diabetic nephropathy.^113^, ^115^ Increased tubule cell senescence in a streptozotocin (STZ)‐induced mouse model of AKI has been linked to elevated levels of TIF markers (e.g., collagen‐1α1, collagen‐14α3, TGFβ‐1 and α‐smooth muscle actin [α‐SMA]),^53^ with administration of the anti‐tumorigenic heat shock protein (HSP)‐90 inhibitor alvespimycin—either alone or in combination with senostatic GS‐444217—reducing senescent cell burden, markers of inflammation (cluster of differentiation 68 [CD68], tumour necrosis factor alpha [TNF‐α], chemokine ligand 2 [CCL2]) and blood urea nitrogen.^53^ Furthermore, in a similar study using STZ mice, elevated levels of senescent tubular cells were associated with increased levels of SASP (e.g., IL‐6 and TNF‐α), and markers of senescence (e.g., p21), events attenuated when the complement component 5a receptor 1 (C5AR1) was deleted either genetically or pharmacologically.^114^ While these studies outline a link between the diabetic microenvironment, senescence and tubular function, beneficial effects of pharmacological agents that intercept across a range of different pathways further highlight the complexity and heterogeneity of these events.

The onset and progression of TIF necessitates the involvement of multiple cell types, namely tubule cells, fibroblasts and infiltrating macrophages. As it is well established that senescent cells exhibit the bystander effect,^61^ their accelerated accumulation in the proximal tubule provides a potential route by which they could orchestrate paracrine‐mediated cell‐to‐cell crosstalk. Chronic accumulation of renal tubular senescent cells in in vivo models of kidney disease leads to persistent and sustained release of SASP factors that can trigger fibroblast activation leading to maladaptive kidney repair and TIF.^91^, ^116^ Selective clearance of these cells is associated with a reduction of renal fibrosis and improved tubule cell regeneration and function, evidenced by restoration of GFR.^91^ In support of these observations, pro‐fibrotic and inflammatory proteins secreted by senescent PTECs drive activation and proliferation of fibroblasts in a high glucose in vitro environment.^116^ Reinforced by Fu et al.^55^ this suggests that in the face of glycaemic injury, stress‐induced senescence of PTECs may represent a notable biological event in the progression of DKD.^55^

## 5 MECHANISMS OF CELLULAR SENESCENCE IN THE KIDNEY

**FIGURE 2:**
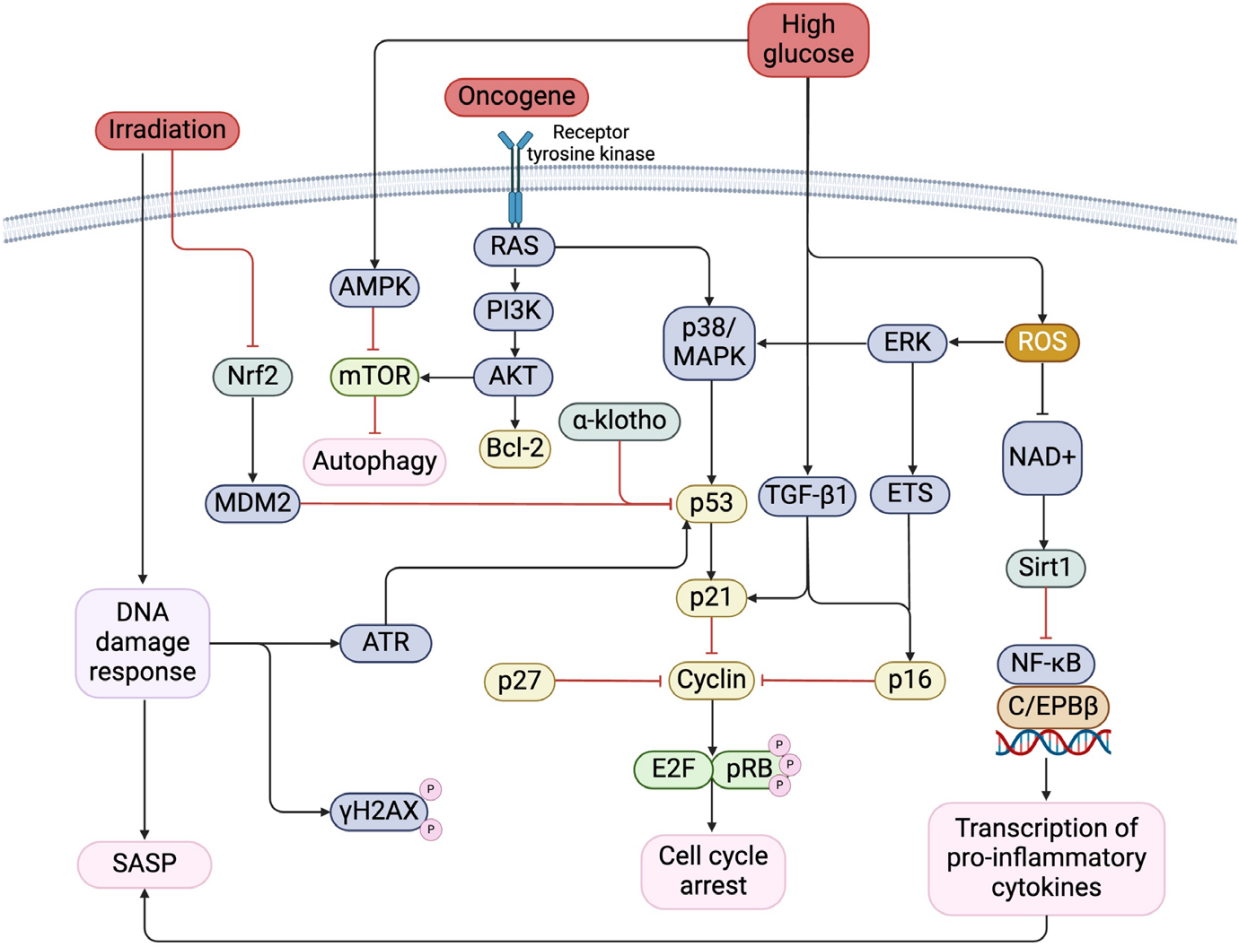
Multiple pathways govern the induction of senescence and ultimately culminate in the phosphorylation of pRB to induce cell cycle arrest. Sustained hyperglycaemia triggers up‐regulation of reactive oxygen species and pro‐fibrotic cytokine TGFβ‐1, leading to increased transcription of pro‐inflammatory cytokines and cell cycle arrest. High glucose can also regulate AMPK and mTOR activity, leading to repression of autophagy which promotes senescence in its later stages. Similarly, oncogene activation culminates in repression of autophagy through modulation of the PI3K/AKT axis. This pathway directly regulates mTOR activity to down‐regulate autophagy and promote the senescent state. In addition to effects on autophagy, oncogene activation also induces cell cycle arrest through the p38/MAPK axis, which leads to p53 up‐regulation and consequent arrest via inhibition of cyclins. Exposure to radiation also causes up‐regulation of p53 through effects on Nrf2 activity and MDM2. AKT, protein kinase B; AMPK, adenosine monophosphate‐activated protein kinase; ATM, ataxia‐telangiectasia mutated; C/EPB β, CCAAT‐enhancer‐binding protein beta; ERK, extracellular signal‐regulated kinase; ETS, erythroblast transformation specific; MAPK, mitogen‐activated protein kinase; MDM2, mouse double minute 2; mTOR, mammalian target of rapamycin; NAD, nicotinamide adenine dinucleotide; NF‐kB, nuclear factor kappa B; Nrf2, nuclear factor erythroid 2‐related factor; P13K, phosphatidylinositol 3‐kinase; pRB, phosphorylated retinoblastoma; RAS, rat sarcoma; ROS, reactive oxygen species; SASP, senescence‐associated secretory phenotype; TGFβ‐1, transforming growth factor beta 1.

As summarised in Figure 2, several pathways come together to initiate senescence within the diabetic kidney. Understanding how these pathways interact and orchestrate senescence and its SASP enables future identification of therapeutic targets.

### 5.1 The p16/RB and p53/p21 axes

Involved in irreversible cell cycle arrest, the p16/retinoblastoma (RB) and/or p53/p21 pathway targets p16/RB‐induced cell cycle arrest and the release of E2F, a transcription factor that facilitates progression through the cell cycle.^11^ Control of cell cycle inhibition fluctuates, with the involvement of the p53/p21 pathway predominating during initiation of senescence, and the p16/RB arm more prevalent in maintenance of the senescent state.^11^

In the p16/RB axis, p16 inhibits the cyclin‐dependant kinase 4/6‐cyclin D complex, which dephosphorylates RB‐E2F and initiates cell cycle arrest.^117^ Expression and activation of p16 can be attributed to injury caused by oxidative stress^118^ and advanced glycation end products,^119^ both of which correlate with DKD as a consequence of hyperglycaemia. In the p53/p21 axes, p53 becomes phosphorylated and up‐regulates expression of p21 which can inhibit the cyclin‐dependant kinase 2‐cyclin E complex.^11^ This mechanism culminates in subsequent dephosphorylation of RB‐E2F, leading to cell cycle arrest in multiple cell types, including the kidney.^11^ Activation of p53 can occur directly in response to elevated glucose and is markedly increased in renal tubular cells in both in vitro and in vivo models of diabetes.^120^ Similarly, p21 expression is induced in response to hyperglycaemia where tubular levels of p21 are associated with the severity of DKD.^120^

### 5.2 AMPK/mTOR signalling

An adenosine triphosphate (ATP)‐dependant protein kinase, AMPK is an essential protein which supplies energy for use in normal/healthy cellular activities.^121^ Activated in response to various stimuli, for example, hypoxia and nutritional deficiency,^121^ AMPK has several roles in ageing, including inhibition of mTOR, a potent inhibitor of autophagy and consequent promotor of senescence.^122^ Inhibition of mTOR delays senescence in several cell types,^121^, ^123^ suggesting negative crosstalk between these two proteins. This interrelationship can be observed in renal PTECs, where inhibition of mTOR and activation of AMPK reduces hyperglycaemia‐induced senescence.^124^ Conversely, elevated glucose significantly increases mTOR expression, activity, and associated senescence in mesangial cells.^125^ These data suggest a functional role for AMPK and mTOR in the induction of renal senescence, where compounds targeting their activity may delay age‐related diseases, for example, rapamycin has shown promising results in suppressing cellular senescence^125^ and improving renal tubular injury in vitro.^125^

### 5.3 Autophagy

Autophagy is a highly conserved eukaryotic process, defined as a lysosomal degradation pathway which recycles cellular components to maintain energy homeostasis.^126^ Importantly, autophagy deficiency within the kidneys—specifically the proximal tubules—has been observed in people with T2DM^127^ and diabetic nephropathy,^128^ as well as in rodent models of diabetes.^129^, ^130^, ^131^ Induction of autophagy can be attributed to phosphorylation and activation of AMPK,^104^ which regulates the expression of downstream autophagy genes (e.g., forkhead box O3 [FOXO3] and bromodomain‐containing protein 4 [BRD4]), and also promotes autophagy directly by phosphorylating several autophagy‐related proteins.^132^ The inhibitory relationship between autophagy and senescence has been well characterised.^133^, ^134^, ^135^ In in vivo models of STZ‐induced DKD, activation of autophagy attenuates podocyte senescence.^104^ Similarly, selective clearance of senescent cells by senolytics (i.e., dasatinib and quercetin) activates autophagy in podocytes which protects against DKD progression.^100^ Activation of autophagy was also associated with reduced levels of senescence in murine models of both T1DM and T2DM, specifically in three renal cell types when cultured in vitro (i.e. PTECs, mesangial cells and podocytes).^136^ Notably, the use of rapamycin, an inhibitor of mTOR, activated autophagy and reduced levels of p16 and p21 in PTECs.^123^ In contrast, murine models with a renal tubule‐specific autophagy knockout exhibit increased tubular cell senescence, leading to maladaptive kidney repair post‐ischaemic AKI.^137^ Interestingly, mitophagy, the selective degradation of mitochondria by autophagy, has also been linked to induction of cell senescence in primary proximal tubule cells in people with type 2 DKD^138^ and in STZ‐induced mice.^139^

Initially believed to have a one‐dimensional role in negatively regulating cellular senescence through targeting cells for degradation, a recent study suggests autophagy may differentially modulate cellular senescence via a pro‐senescence autophagy‐mediated function. Zhang et al. demonstrated that kidney PTECs exhibit elevated expression of both senescence and autophagy‐related proteins in response to high glucose.^135^ High levels of tubular autophagosomes were also observed, and co‐incubation with an inhibitor of autophagy reversed increases in p21 and p53 expression.^135^ While these results suggest a dual role for autophagic flux in hyperglycaemia‐induced renal tubular senescence, further studies are required to support such a statement.

### 5.4 Wnt signalling

Wingless‐related integration site (Wnt)/β‐catenin signalling comprises a pathway crucial in normal organogenesis and tissue repair in healthy individuals.^140^ In healthy adult kidneys, Wnt signalling is usually silent, but is reactivated in response to renal injury^141^ and the ageing process.^142^ The canonical Wnt/β‐catenin pathway involves nuclear translocation of β‐catenin following dephosphorylation, from where it binds to transcription factor T‐cell factor (TCF)/lymphoid‐enhancer‐binding factor (LEF), triggering up‐regulation of downstream target genes (e.g., fibronectin, collagen I and granulocyte colony stimulating factor).^143^, ^144^

Studies have confirmed the effects of Wnt signalling in promoting cellular senescence in many cell types involved in age‐related disease, including alveolar epithelial cells,^145^ and chondrocytes.^146^ Immunostaining of kidney biopsies from people with various nephropathies, including diabetic nephropathy, revealed that Wnt9a expression was significantly increased and predominantly localised to the renal tubular epithelium.^147^ These changes were positively correlated to elevated expression of senescence markers, such as β‐galactosidase and p16, with ablation of Wnt9a reversing these changes.^147^ Similarly, in a gene set enrichment analysis, senescent cells from the renal epithelium demonstrated significant increases in Wnt signalling when compared with non‐senescent counterparts.^148^

Activation of the Wnt/β‐catenin pathway has been shown to promote tubular cell senescence in murine models of kidney disease.^149^ Here, up‐regulation of senescence through the Wnt/β‐catenin pathway resulted in development of renal fibrosis, with inhibition of this pathway negating increases in markers of senescence.^149^ Ectopic expression of Wnt1 was also associated with increases in cellular senescence in PTECs,^142^ with inhibition of Wnt/β‐catenin restoring these changes.^142^ Furthermore, a study by Luo et al. reported that the Wnt9a ligand promotes accelerated cellular senescence of PTECs in rodent models of kidney injury.^147^ Consequently, aberrant Wnt/β‐catenin signalling has been identified as a key mediator of cellular senescence within the kidney.

## 6 ENDOGENOUS PROTECTION AGAINST CELLULAR SENESCENCE WITHIN THE KIDNEY

**FIGURE 3:**
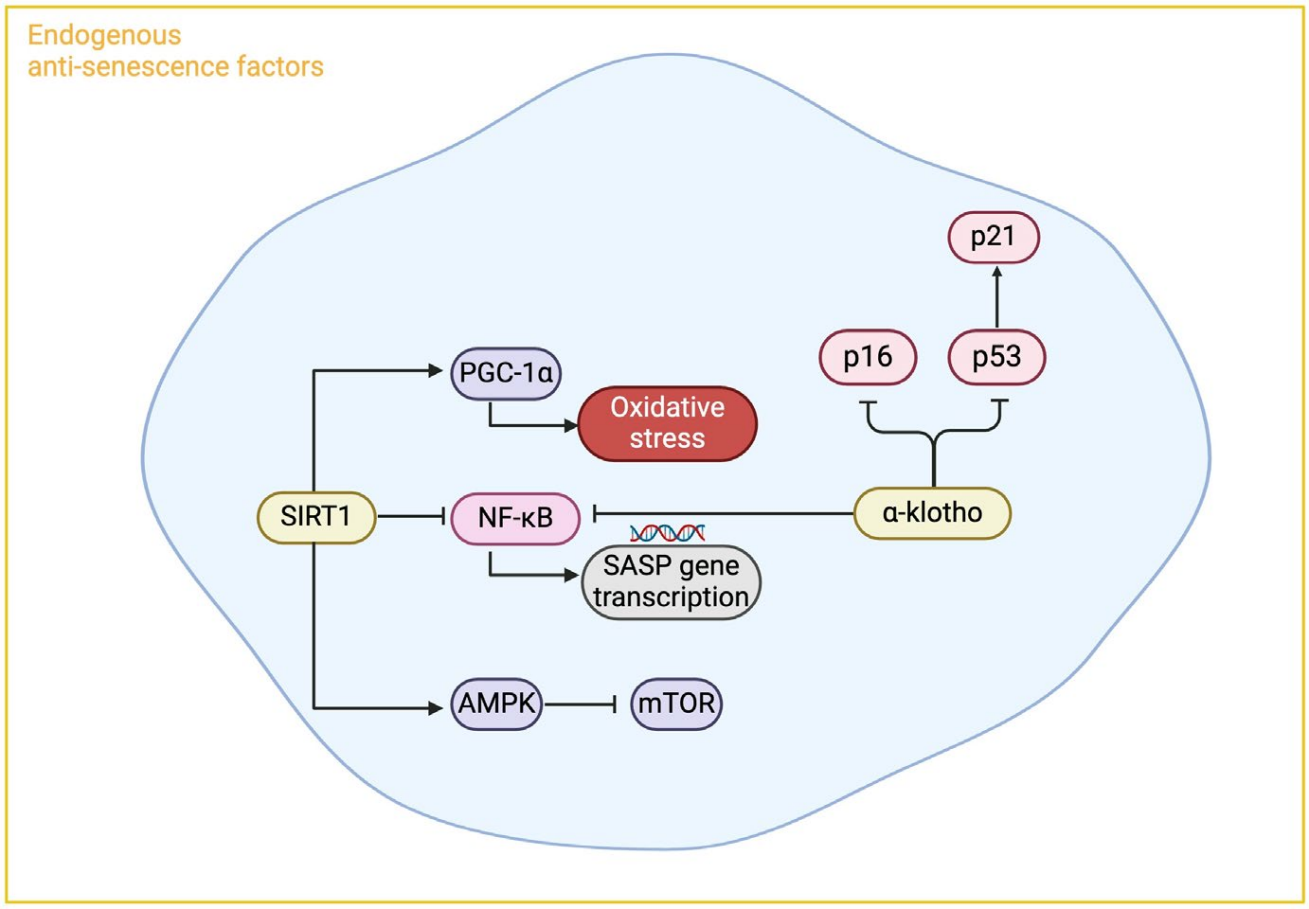
Endogenous factors and their mechanisms of action to protect against cellular senescence. Endogenous factors SIRT1 and α‐klotho have been shown in studies to have anti‐senescence effects through mediation of several pathways implicated in the induction of cellular senescence. SIRT1 is a negative regulator of NF‐kB, a potent transcription factor associated with regulation of SASP gene transcription, and also has regulatory effects on AMPK and PGC‐1α, Similarly, α‐klotho can repress NF‐kB activity and directly reduce senescence through effects on cell cycle inhibition. AMPK, adenosine monophosphate‐activated protein kinase; mTOR, mammalian target of rapamycin; NF‐kB, nuclear factor kappa B; PGC‐1α, peroxisome proliferator‐activated receptor‐gamma coactivator‐1α; SASP, senescence‐associated secretory phenotype. Peroxisome proliferator‐activated receptor‐gamma coactivator‐1α.

Due to the heterogeneous nature of senescence and its dual role in both longevity and disease, the process is dichotomously controlled by both negative and positive regulators. Importantly, several endogenous factors are involved in the regulation of cellular senescence within the kidney and are thought to naturally protect against progression of DKD (Figure 3).

### 6.1 Klotho

Predominantly expressed in tubular cells, klotho is a pleiotropic protein with both reno‐protective and gero‐protective (anti‐ageing) functions within the kidney.^150^ It acts as an endocrine signalling molecule and has multiple protective roles through its ability to dampen oxidative stress, extend lifespan and improve insulin sensitivity.^151^ Studies have identified the relative loss of soluble klotho in the plasma as a predictor of renal impairment in people with T2DM.^152^, ^153^ In this context, loss of klotho negatively correlates to the annual rate of decline in estimated GFR in people with diabetes,^152^ suggesting it may be a useful biomarker for predicting renal impairment in this group of people. Deficiency in soluble klotho is also associated with microalbuminuria in individuals with T1DM,^154^ suggesting a causal role for klotho deficiency on albumin excretion, while recent observations associate diminished klotho with albuminuric DKD in people with T2DM.^155^

In mouse models of obesity, selective clearance of senescent cells using the senolytics dastatinib and quercetin restored klotho levels within the kidney, highlighting an inverse relationship between klotho and cellular senescence.^156^ This inhibitory relationship has been documented in studies using models of kidney disease^157^, ^158^ where supplementation of rodent kidney cells with klotho blunted oxidative stress‐induced senescence and reduced cellular injury.^158^ Regulated via a Wnt9a‐dependant pathway, ectopic expression of klotho was able to mitigate increases in p16 protein expression and SA‐β‐gal activity in murine models of CKD.^157^ Similarly, klotho abolished renal fibrosis in a high glucose‐induced accelerated ageing murine model through antagonist effects on endogenous Wnt signalling, a well established pathway in the induction of renal senescence and a potential pathway in which klotho may mediate its anti‐senescence effects.^128^ In support of this suggestion, overexpression of klotho in murine models of T2DM abolished injury in renal glomerular endothelial cells while also negating increased Wnt‐β‐catenin signalling.^151^ Comparatively, regulation of autophagy may be another pathway by which klotho protects against cellular senescence, since autophagy is a recognised repressor of senescence induction. A study by Xue et al. confirmed that klotho expression was down‐regulated in murine models of T1DM and high glucose‐induced PTECs; however, overexpression of klotho was associated with enhancement of autophagy and AMPK both in vivo and in vitro.^159^ Soluble klotho also increased renal levels of AMPK in in vivo models of T2DM while down‐regulating levels of mTOR.^160^

The anti‐inflammatory properties exerted by klotho are also thought to confer renoprotection in response to disease. Exogenous supplementation of klotho supresses cytokine production following TNFα stimulation in murine models of CKD.^161^ In rodent renal cells exposed to oxidative stress‐induced senescence, treatment with klotho abrogates increases in SASP markers (e.g., IL‐6, TNFα and IL‐1β).^158^ Further studies are needed to evaluate a mechanistic role for klotho in mediating renal senescence in the presence of glycaemic injury.

### 6.2 Sirtuin1

Sirtuin1 (SIRT1) is a member of a conserved family of nicotinamide adenine dinucleotide (NAD+)‐dependant deacetylases that exert a wide range of cellular functions in ageing and cellular homeostasis.^162^ Moreover, with potent antioxidant properties, SIRT1 protects against oxidative stress, a recognised hallmark of age‐associated conditions, including DKD. In fact, loss of SIRT1 has been identified as a biomarker of DKD,^163^ with renal levels reportedly decreased in murine models of both T1DM and T2DM.^164^ In murine models of overfeeding, decreased SIRT1 is associated with induction of renal cellular senescence, confirmed by increases in SA‐β‐gal activity and elevations in cell cycle inhibitor p53.^165^ Studies have demonstrated that SIRT1 is able to combat oxidative stress by modulating transcriptional activities of proteins involved in the oxidative stress response. Specifically, SIRT1 has been shown to be a regulator of peroxisome proliferator‐activated receptor‐gamma coactivator‐1α (PGC‐1α), a transcriptional factor which prevents and protects murine podocytes against oxidative stress.^166^ Consequently, SIRT1 can reduce high glucose‐induced oxidative stress within the kidney, attenuating progression of DKD in STZ mice.^167^ Through p53 deacetylation, SIRT1 has also demonstrated attenuation of renal senescence in PTECs.^168^ Furthermore, in murine models of AKI, SIRT1 activation promotes autophagy,^169^ while pharmacological inhibition of SIRT1 (via EX‐527) blocked autophagy in STZ rats having received pharmacological intervention.^170^ In support of this, SIRT1 down‐regulation blocks mesenchymal stem cell‐mediated enhancement of podocyte autophagy in DKD rats.^171^

Not surprisingly, activators of SIRT1, for example, resveratrol, show promising results in arresting high glucose‐induced kidney cell senescence,^125^ with recent findings in aged mice reporting that resveratrol protects against glomerulosclerosis through SIRT1‐mediated klotho expression.^172^ Collectively, these studies highlight SIRT1 as a promising therapeutic target in mediation of renal senescence. However, further studies are now required to delineate a specific role for SIRT1 in the induction of senescence within the diabetic kidney.

## 7 PHARMACOLOGICAL INTERVENTIONS PROMOTING RENAL SENESCENT CELL CLEARANCE

**FIGURE 4:**
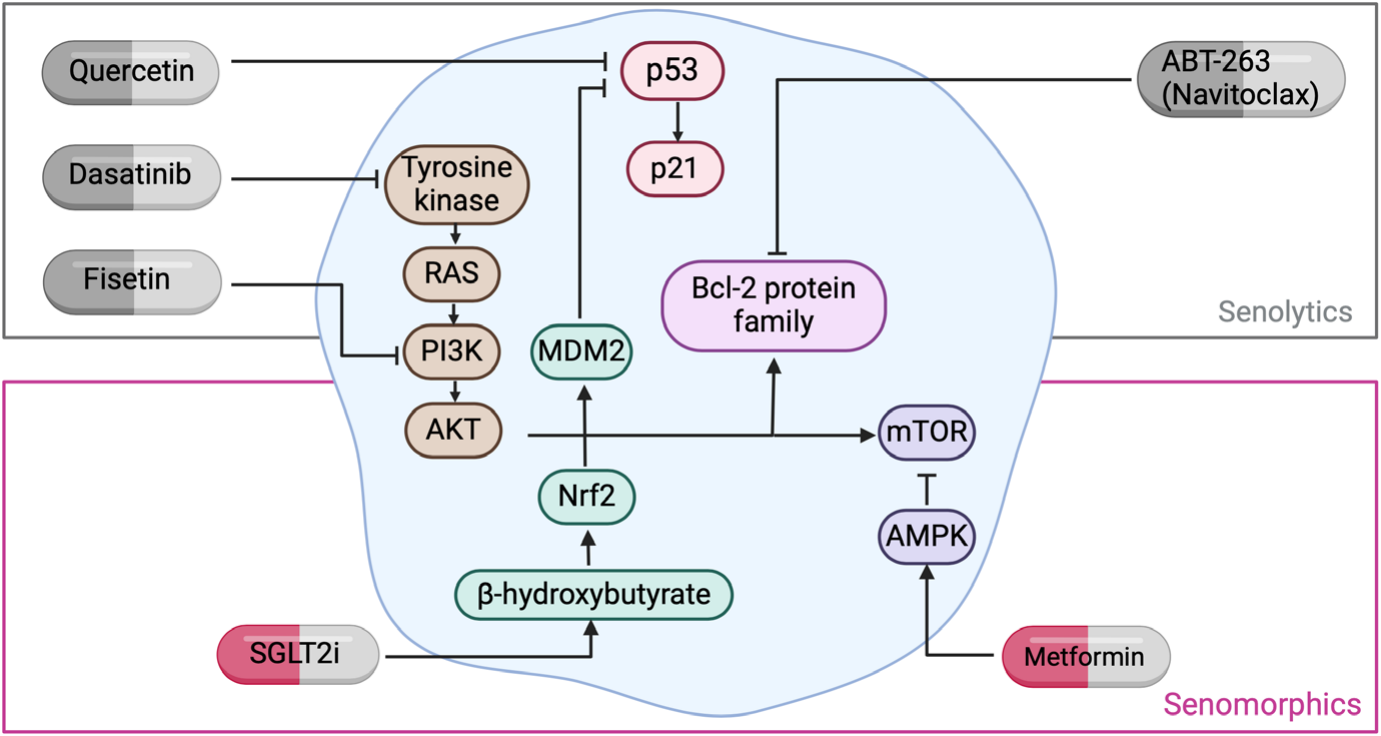
The mechanisms of action of senolytics and senomorphics and their roles in reducing the burden of senescent cells. Senolytics, for example, dasatinib, quercetin, fisetin and navitoclax (ABT‐263) act by initiating pro‐apoptotic pathways (e.g., Bcl‐2) dysregulated by senescence to promote senescent cell clearance. Senomorphics, such as SGLT2is and metformin, work to modulate the pathways involved in senescent cell initiation, thereby targeting them for removal. AKT, protein kinase B; AMPK, adenosine monophosphate‐activated protein kinase; MDM2, mouse double minute 2; mTOR, mammalian target of rapamycin; Nrf2, nuclear factor erythroid 2‐related factor; P13K, phosphatidylinositol 3‐kinase; RAS, rat sarcoma.

Pharmacological interventions that promote the selective clearance of senescent cells are of key therapeutic interest for treatment of DKD. These compounds, referred to as senotherapeutics, can be divided into senolytics (which selectively eliminate senescent cells) and senomorphics (which supress and modulate expression of the SASP). Senolytic therapies are able to overcome the resistance of senescent cells to apoptosis by inducing programmed cell death.^15^ Comparatively, senomorphics modulate the SASP through targeting signalling pathways linked to SASP expression^173^ (Figure 4).

### 7.1 Dasatinib, quercetin and fisetin

Dasatinib is a second‐generation tyrosine kinase inhibitor approved for the treatment of chronic myeloid leukaemia, while quercetin is a plant flavonoid abundant in several fruits and vegetables that possesses antioxidant and anti‐inflammatory properties.^100^ The therapeutic potential of dastatinib and quercetin in eliminating the senescent cell burden in disease has been evaluated in several in vivo and in vitro studies. In diabetic mice, dastatinib and quercetin enhanced renal function and improved histopathological changes, including a reduction in renal fibrosis and glomerular basement membrane expansion.^100^ In this context, the benefits of dastatinib and quercetin are believed to be mediated by the activation of autophagy.^100^ Similarly, dastatinib and quercetin abrogated the senescence response in renal PTECs exposed to the diabetic microenvironment, and attenuated changes in expression of p16, p53 and fibronectin.^113^

The use of dastatinib and quercetin in reducing total senescent cell burden is currently under investigation in an open‐label, phase I pilot study (NCT02848131).^76^ Study participants include adults aged 50–80 years with diabetes mellitus (on anti‐diabetes therapy) and CKD (estimated GFR range: 15–45 mL/min/1.73 m^2^).^76^ Preliminary observations report decreased expression of p21 and p16 and a reduction in senescent cell accumulation as evidenced by SA‐β‐gal activity in adipose tissue biopsies isolated from these individuals (11 days post‐drug initiation).^76^ Circulating SASP factors (such as IL‐6, MMP‐9, MMP‐12 and IL‐1α) were also reduced, as was accumulation of CD68^+^ tissue macrophages,^76^ the latter of which was attributed to decreased macrophage attraction and loss of anchoring within adipose tissue as a consequence of senescent cell clearance. Despite promising results, it is important to note that one of the major limitations dastatinib and quercetin is their potential for nephrotoxicity.^174^, ^175^ While pre‐clinical and clinical trials have not provided conclusive evidence of these toxic effects, real‐world applications of dasatinib have been associated with rare renal adverse effects (e.g., proteinuria and diffuse foot process damage).^174^ Consequently, at present, the potential for clinical application of dastatinib and quercetin remains limited, with further studies now required to assess any potential off‐target effects.

Similar to quercetin, fisetin is a plant flavonoid which possesses a variety of pharmacological properties, such an antioxidant, anti‐inflammatory and anti‐cancer activities.^176^ The therapeutic potential of fisetin to protect against senescence‐related changes in the kidney has recently been reported. In in vitro models of high glucose‐induced podocyte injury, treatment with fisetin attenuates a glucose‐induced loss of function in podocytes, effects which were paralleled by suppression of the NLRP3 inflammasome and increased autophagy, the latter evidenced by increased autophagosome formation.^73^, ^177^, ^178^ In murine models of CKD^91^, ^179^ and T1DM^176^ fisetin administration reduced senescent cell burden and renal fibrosis, culminating in improved tubular function and improved kidney injury.^91^ Notably, the anti‐fibrotic effects of fisetin have been attributed to inhibition of the TGF‐β1 pathway;^91^, ^176^ a ubiquitous cytokine well documented as a driver of both senescence and renal fibrosis in the context of hyperglycaemia.^94^, ^95^, ^180^ Through phosphorylation of signalling intermediates (Smad 2/3), fisetin reduced protein expression of extracellular matrix components (e.g., α‐SMA) and attenuated increases in fibrosis‐related genes (e.g., collagen 1).^176^

Although in vitro and in vivo results of fisetin treatment on alleviating senescent cell burden in the diabetic kidney are promising, evaluation of fisetin in a clinical trial setting has yet to be conducted, with little information currently available regarding long‐term side effects or health risks of drug use.

### 7.2 ABT‐263 (Navitoclax)

As an orally active Bcl‐2 inhibitor, ABT‐263 disrupts Bcl‐2/Bcl‐xL interactions with pro‐apoptotic proteins, triggering the initiation of apoptosis.^12^ Currently only tested in euglycaemic conditions, ABT‐263 selectively eliminates senescent cells within the proximal tubular epithelium of young and aged mice with AKI, reducing fibrosis and improving renal function.^181^ Similar improvements are observed in a model of CKD, where ABT‐263 attenuates renal fibrosis and improves tubular repair after repeated treatment with cisplatin.^91^ Although not evaluated in DKD, the ability of ABT‐263 to reduce senescent cell burden makes it a compound of interest for future investigations. However, as inhibition of Bcl‐xL has effects on platelet survival, ABT‐263 has been linked with thrombocytopenia.^182^ Therefore, similar to dastatinib and quercetin, the therapeutic potential of ABT‐263 remains to be determined.

### 7.3 Metformin

Metformin is commonly used as a first‐line treatment of T2DM.^183^ However, its efficacy in regulating cellular and metabolic processes involved in the development of age‐related diseases has reignited widespread interest beyond its glycaemic actions. In computational modelling, metformin was shown to directly activate SIRT1,^184^ with studies reporting that metformin reduced glucose‐induced cell senescence in PTECs as evidenced by diminished p21 mRNA expression and β‐gal staining.^56^ These findings were corroborated in rodent models of T2DM, where metformin improved senescent cell burden in the tubular epithelium in response to a high‐glucose microenvironment.^56^, ^183^ The protective effects of metformin in this context are thought to be mediated through the AMPK/SIRT1‐FoxO1 axis, which works to reduce oxidative stress, a potent inducer of senescence, while enhancing autophagy.^185^

Effects of metformin in attenuating the hallmarks of ageing are being evaluated in the ongoing ‘targeting ageing by metformin (TAME)’ clinical trial, which examines the efficacy of metformin in delaying the onset of age‐related diseases by modulating mechanistic pathways, for example, cellular senescence.^186^ As metformin has already exhibited favourable effects in reducing senescent cell burden in both in vitro and in vivo studies, its translational potential as an anti‐ageing therapy is apparent and may play a crucial role in the treatment landscape for DKD.

### 7.4 Sodium/glucose cotransporter‐2 (SGLT2) inhibitors

Sodium‐glucose co‐transporter 2 inhibitors (SGLT2is, e.g., empaglifozin, dapaglifozin and canagliflozin) are glucose‐lowering drugs currently prescribed for the treatment of T2DM.^187^, ^188^ They selectively target the SGLT2 membrane protein in the proximal tubule, preventing glucose reabsorption, while preserving GFR through increased tubuloglomerular feedback and decreased hyperfiltration.^189^ In addition, SGLT2is have demonstrated adjunct protective effects outside of their glucose lowering ability, with improved cardiovascular and renal outcomes observed in the absence of diabetes.^190^ However, the mechanism by which this occurs remains to be elucidated.

It has been proposed that SGLT2is may target the ageing process itself,^191^ with recent studies highlighting a role for the SGLT2 protein in kidney senescence, and hyperglycaemia having been observed to induce cellular senescence in DKD via an SGLT2‐dependant pathway.^54^, ^87^, ^192^, ^193^ The diabetic microenvironment has been shown to increase expression of SGLT2 within proximal tubule epithelial cells in in vitro models of diabetes.^57^, ^115^ Comparatively, inhibition of SGLT2 is associated with reduced tubular senescence in STZ‐induced mice,^57^ and in in vitro models of diabetic nephropathy.^115^, ^192^ The protective effects of SGLT2is in amelioration of senescence are likely due to their roles in reducing oxidative stress and DNA damage through induction of antioxidant pathways.^192^ Regulation of autophagy has been proposed as one of the mechanisms by which SGLT2is reduce cellular senescence and protect against DKD progression. In PTECs, empagliflozin was shown to increase AMPK levels and recover autophagic flux, as evidenced by increased formation of autophagosomes in the presence of high glucose.^194^ In vivo, these results were corroborated in murine models of T2DM displaying reactivation of glomerular autophagy after treatment with empagliflozin.^131^ Moreover, dapagliflozin confers protective effects in T2DM murine models of DKD through increased AMPK activity,^195^ events in vivo linked to restoration of autophagy in high glucose‐treated PTECs.^196^ Similarly, hyperactivation of mTOR is reversed after treatment with empagliflozin in both in vitro models of diabetes^194^ and in vivo models of DKD.^193^ As increased mTOR activity is a hallmark of senescence and potent inhibitor of autophagy, SGLT2‐mediated inhibition of mTOR may present a viable mechanism by which SGLT2is suppress senescence and confer renoprotection.^57^, ^192^, ^193^

## 8 CONCLUSION

The pathogenesis of DKD is driven by chronic and dysregulated inflammation and fibrosis. Notably, the aberrant accumulation of senescent cells has been implicated in this process, with many studies demonstrating the deleterious effects of cellular senescence in diabetes‐associated nephropathy.^56^, ^112^, ^114^, ^197^ Exposure to various stress‐inducing stimuli (e.g., hyperglycaemia), promotes cellular senescence and inhibits damage repair and regeneration within the kidney. Subsequently, the release of pro‐inflammatory and pro‐fibrotic molecules through the senescent secretome results in a perpetual cycle which further perpetuates DKD progression. Reno‐protective benefits of senomorphics and senolytics have been assessed in various models of kidney disease. Repurposing currently approved therapeutics for diabetes management (e.g., metformin and SGLT2i) for modulation in the pathology of age‐related disease offers promising therapeutic potential. However, to generate future pharmacological therapies which prevent disease progression with minimal contraindications, future research is needed to help better understand the specific molecular mechanisms of senescence in DKD.

## CONFLICT OF INTEREST STATEMENT

The authors have no conflict of interest to declare.

## Data Availability

Data sharing is not applicable to this article as no new data were created or analyzed in this study.
